# Risk Factors and Outcomes of Acute Pulmonary Embolism in African Patients: A Systematic Review

**DOI:** 10.7759/cureus.74673

**Published:** 2024-11-28

**Authors:** Collins C Okeke, Emmanuel S Amadi, Onyinye E Ebiliekwe, Ifunanya R Ekeocha, Emeka Nnanna Okoro, Oluchi J Nduji, Malipeh-Unim Undie, Onyinye Ngige, Anthony Eze-odurukwe, Chinecherem Ezema, Afamefuna Onyeogulu, Angela Ojo, Michael Obuseh, Kelechi Okonta

**Affiliations:** 1 Department of Internal Medicine, University of Port Harcourt Teaching Hospital, Port Harcourt, NGA; 2 Department of Internal Medicine, Hallel Hospital and Maternity, Port Harcourt, NGA; 3 Department of Internal Medicine, Nnamdi Azikiwe University Teaching Hospital, Nnewi, NGA; 4 Department of Internal Medicine, Imo State University College of Medicine, Owerri, NGA; 5 Department of Paediatrics, Abia State University, Uturu, NGA; 6 Department of Anaesthesiology, Surgery Interest Group of Africa, Lagos, NGA; 7 Department of Surgery, Salford Royal NHS Foundation Trust, Manchester, GBR; 8 Department of Internal Medicine, Afe Babalola University, Ado Ekiti, NGA; 9 Department of Internal Medicine, Delta State University Teaching Hospital, Oghara, NGA; 10 Cardiothoracic Surgery Unit, Department of Surgery, University of Port Harcourt Teaching Hospital, Port Harcourt, NGA

**Keywords:** deep vein thrombosis, outcome, pulmonary embolism, risk factors, venous thromboembolism

## Abstract

Pulmonary embolism is a common cause of morbidity and mortality. Numerous risk factors have been identified that predispose patients to this disease. This study aims to identify these risk factors and the possible outcomes (recovery or mortality) after receiving treatment from any hospital. Healthcare is expensive in Africa, hence hindering its easy accessibility.

PubMed, Scopus, and African Journals Online were searched from the database inception to October 2024 to identify relevant studies. A total of 719 articles were identified, for which 172 duplicate articles were removed. After screening 592 articles by title and abstract, 508 were excluded. Eighty-four articles were screened by full text to determine their eligibility. Finally, 13 articles were used in the final qualitative analysis. We included original research published in English in peer-reviewed journals from January 2000 to September 2024 that reported the risk factors and outcomes of pulmonary embolism, and studies that used computed tomography pulmonary angiography as a diagnosis of acute pulmonary embolism in patients more than 18 years old, irrespective of gender and medical or surgical condition, managed in any African hospital, were included.

In total, 7650 patients were included in 13 articles, from 10 countries (Nigeria, Togo, Angola, Kenya, Cameroon, South Africa, Sierra Leone, Egypt, DR Congo, and Ethiopia), and 861 patients had pulmonary embolism. The mean age of the reported patients ranged from 40.8 to 64.4 years across the studies. There were 309 male and 552 female patients diagnosed with pulmonary embolism. The study types included in this review are retrospective studies, cross-sectional studies, and case-control studies. Deep vein thrombosis (DVT), heart disease, immobilization, obesity, smoking, recent surgery, and malignancy were the most commonly identified risk factors across the included articles.

Pulmonary embolism contributes significantly to morbidity and mortality among African patients, with key risk factors including DVT, immobilization, heart disease, obesity, smoking, recent surgery, malignancy, pregnancy, and contraceptive use. Limited diagnostic resources in low-resource settings pose a major challenge, but adopting affordable diagnostic alternatives and clinical algorithms could improve outcomes by enabling earlier diagnosis and timely treatment. The availability and implementation of a standardized PE treatment protocol will ensure quality care, decrease mortality, and increase recovery rates.

## Introduction and background

Pulmonary embolism (PE) and venous thromboembolism (VTE) are the third most common causes of cardiovascular death after myocardial infarction (MI) and cerebrovascular disease [1]. PE results when a thrombus migrates from the venous circulation to the pulmonary vasculature and lodges in the pulmonary arterial system; this occlusion impairs gas exchange and circulation [2]. Unfortunately, PE may be asymptomatic or present with sudden death. Characteristic signs and symptoms such as tachycardia, dyspnea, chest pain, hypoxemia, and shock are non-specific. They are present in many other conditions, such as acute MI, congestive heart failure, or pneumonia [1]. 

VTE is a major worldwide burden of disease with 10 million cases per year and is associated with substantial morbidity and mortality. The incidence of PE is unknown, but in the United States, it is estimated that nearly a third of hospitalized patients are at risk of developing VTE and up to 600,000 cases of VTE are diagnosed per year with 100,000 deaths related to these diseases. In the United States, the estimated incidence of diagnosed VTE is 117 per 100,000, but the true incidence is likely to be higher as these diseases are frequently undiagnosed or diagnosed only at autopsy [2]. Globally, about 234 million major surgical interventions are performed each year, and PE is one of the major postoperative complications; studies have shown an increase in the risk of PE by fivefold [3]. Data were obtained from 123 countries covering a total population of 2,602,561,422. Overall, 50 (40.6%) were European, 39 (31.7%) American, 13 (10.6%) Eastern Mediterranean, 13 (10.6%) Western Pacific, three (2.4%) Southeast Asian, and two (1.6%) African. Of 116 countries classifiable according to population income, 57 (49.1%) were high income, 42 (36.2%) were upper‐middle income, 14 (12.1%) were lower‐middle income, and three (2.6%) were low income. A total of 18,726,382 deaths were recorded, of which 86,930 (0.46%) were attributed to PE [4]. African cohorts comprise only a small percentage of studies published worldwide and are underrepresented in global health reports. This can be due to the unavailability of a record database in Africa.

Various conditions lead to the generation of VTE. Virchow's triad of hypercoagulability, venous stasis, and vessel wall injury provides a model for understanding many of the risk factors. These factors are usually either inherited or acquired. Overall, major risk factors for thromboembolic events include recent immobilization, myocardial ischemia, cerebrovascular accident, surgery, and recent trauma. Additional major risk factors include prior VTE, advanced age, malignancy, known thrombophilia, and indwelling venous catheter. Moderate risk factors include family history of VTE, use of estrogen or hormone replacement therapy, smoking, pregnancy, and obesity [1].

The evaluation largely depends on the likelihood of PE and the stability of the patient. There are scoring systems to assist in the determination of the likelihood of PE and thromboembolic events. Diagnostic scoring systems such as the Wells criteria and Geneva score are often used. However, the PE rule-out criteria (PERC) can help rule out PE in low-risk emergency department patients [1]. Other diagnostic tools include the D-dimer blood test, cardiac enzymes (creatine kinase (CK), CK-myocardial band (MB), cardiac-specific troponin), computed tomography pulmonary angiography (CTPA), pulmonary ventilation/perfusion (V/Q) scan, pulmonary angiography, magnetic resonance imaging (MRI), chest X-ray of the heart and lungs, electrocardiogram, echocardiogram, and duplex ultrasound [5].

Treatment of PE depends on the patient's hemodynamics and focuses on keeping the blood clot from getting bigger and preventing new clots from forming. Prompt treatment is essential to prevent serious complications or death. Treatment can include medicines (blood-thinning medicines called anticoagulants, which prevent existing clots from getting bigger and new clots from forming while your body works to break up the clots, and clot dissolvers, i.e., thrombolytics and fibrinolytics), surgery via the use of a flexible catheter to remove the clot and placement of a vein filter, and embolectomy [1,6]. Complications of PE include the following: sudden cardiac death, obstructive shock, pulseless electrical activity, atrial or ventricular arrhythmias, secondary pulmonary arterial hypertension, cor pulmonale, severe hypoxemia, right-to-left intracardiac shunt, lung infarction, pleural effusion, paradoxical embolism, heparin-induced thrombocytopenia, and thrombophlebitis [7]. This research aims to identify the risk factors predisposing an African patient to PE and the possible outcome (recovery or mortality) after receiving treatment from any hospital. Healthcare is expensive in Africa, hence hindering its easy accessibility. Good knowledge of the risk factors and outcomes of PE will help create awareness and educate members of the community.

## Review

Methods

This systematic review was conducted in accordance with the Preferred Reporting Items for Systematic Reviews and Meta-Analyses (PRISMA) extension for systematic reviews [8]. The study protocol was registered with the International Prospective Register of Systematic Reviews (PROSPERO): CRD42024605634.

Inclusion Criteria

We included original research published in English in peer-reviewed journals from January 2000 to September 2024 that reported the risk factors and outcomes of PE. CTPA was used to confirm the diagnosis of acute PE in patients more than 18 years old, irrespective of gender and medical or surgical condition, managed in any African hospital. Outcomes ranged from recovery to morbidity or mortality.

Exclusion Criteria

Patients under 18 years of age with acute PE not confirmed with CTPA, managed in a hospital outside Africa, and with any other thrombotic event, including but not limited to fat, air, tumor cell, or septic embolism, and research published in another language were excluded. Study designs such as case reports, audits, opinions, reviews, meta-analyses, comments, and editorials were excluded.

A comprehensive search was conducted on PubMed, Scopus, and African Journals Online (AJOL) from inception to October 2024. The keywords used were (pulmonary embolism) AND (Africa) and "pulmonary embolism" "Africa", as shown in Appendix A. 

Duplication, title, and abstract screening were performed by three independent reviewers (C.C.O., E.S.A., O.E.E.) against the predefined eligibility criteria using the Rayyan systematic review software (Rayyan Systems Inc., Cambridge, MA, USA). Potentially eligible studies were screened for full-text review. Disagreements were discussed among reviewers; in the case of no resolution, an appeal was made to another reviewer (I.R.E.).

We extracted data from articles related to the author, study year, study design, sample size, number of patients with PE, mean age, gender, risk factors for PE, diagnostic criteria, treatment offered, and outcome of PE. The risk of bias in the included studies was assessed using the Joanna Briggs Institute (JBI) critical appraisal tool for cohort, cross-sectional, and case-control studies. JBI's critical appraisal tools assist in assessing the trustworthiness, relevance, and results of published papers. The purpose of this appraisal is to assess the methodological quality of a study and to determine the extent to which a study has addressed the possibility of bias in its design, conduct, and analysis [9]. Articles are assessed with a yes, no, not clear, and not applicable as shown in Appendix B, Appendix C, and Appendix D.

Results

**Figure 1:**
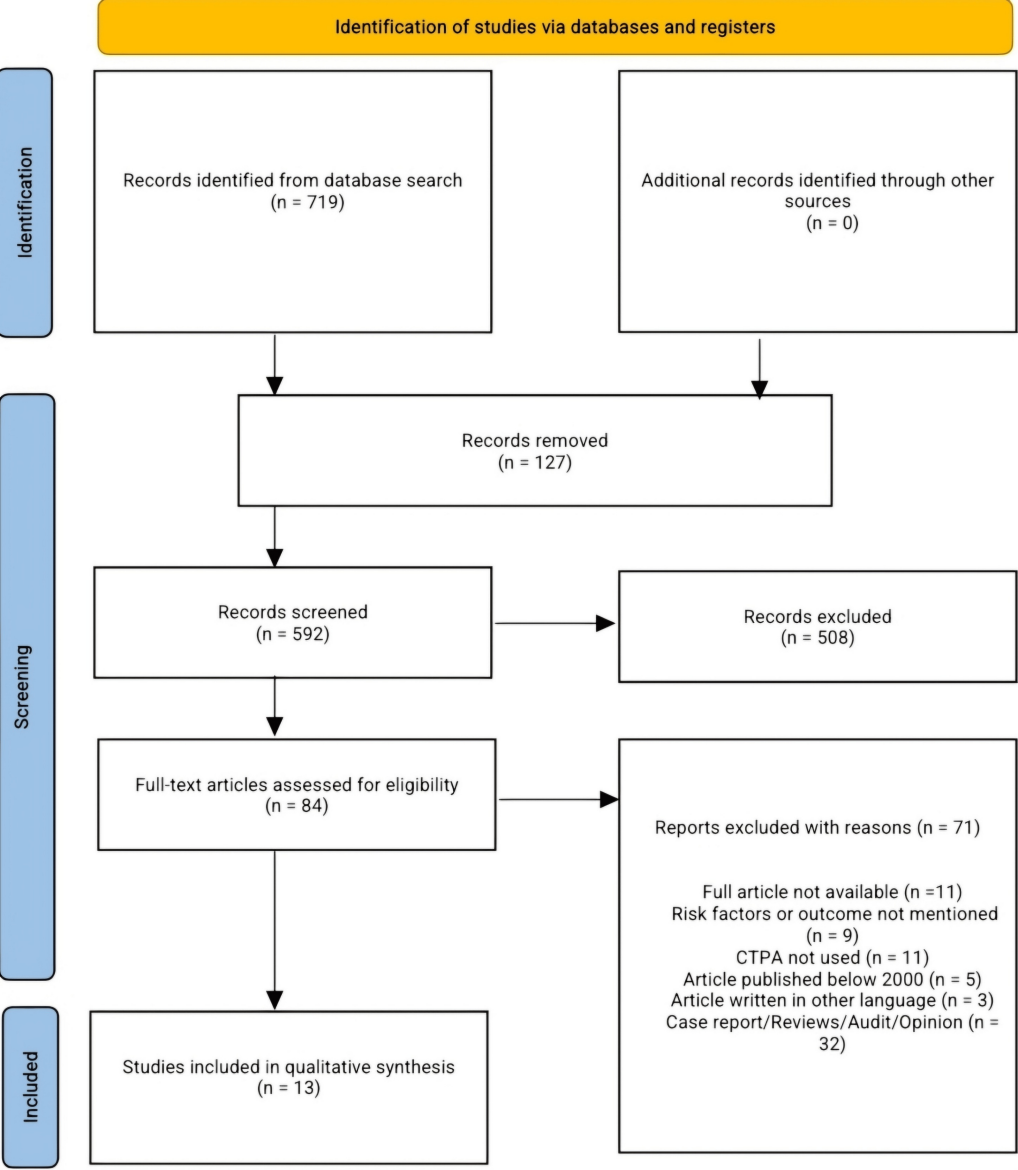
PRISMA flow diagram. PRISMA: Preferred Reporting Items for Systematic Reviews and Meta-Analyses; CTPA: computed tomography pulmonary angiography

Our search returned 719 articles, of which 592 were screened by title and abstract after duplicates were removed. Following the title and abstract screening, 508 articles were excluded, and 84 articles were subjected to full-text screening to determine their eligibility based on our inclusion criteria. Ultimately, 13 articles were included in the final qualitative synthesis. Exclusions were made due to various reasons, including failure to meet our inclusion criteria, unavailability of full articles, risk factors or outcomes of PE not mentioned, CTPA not used as one of the diagnostic criteria, an article published below the year 2000, and an article written in other languages (French language). Figure 1 displays the PRISMA flow diagram.

In total, 7650 patients were included in 13 articles, from 10 countries (Nigeria, Togo, Angola, Kenya, Cameroon, South Africa, Sierra Leone, Egypt, DR Congo, and Ethiopia), and 861 patients had PE. The study period ranged from 2011 to 2023. The mean age of the reported patients ranged from 40.8 to 64.4 years across the studies. There were 309 male and 552 female patients diagnosed with PE. The study characteristics of the included article are listed in Table 1 below.

**Table 1:** Study characteristics. PE: pulmonary embolism

Author	Year	Country	Sample size	Sample size with PE	Mean age	Male	Female
Ogunkoya et al. [10]	2020	Nigeria	31	31	55.1	9	22
Pessinaba et al. [11]	2015	Togo	1622	51	52.7	16	35
Manuel et al. [12]	2015	Angola	50	50	50.5	26	24
Ogeng'o et al. [13]	2011	Kenya	128	128	40.8	60	68
Tambe et al. [14]	2011	Cameroon	37	12	47.6	7	5
Bulajic et al. [15]	2015	South Africa	127	41	45	13	28
Meel et al. [16]	2013	South Africa	498	147	46.8	32	115
Russell et al. [17]	2019	Sierra Leone	4181	79	64.1	35	44
Hussein et al. [18]	2023	Egypt	297	54	55.9	9	45
Bakebe et al. [19]	2014	DR Congo	158	58	54.8	32	26
Raghubeer et al. [20]	2019	South Africa	301	81	43	20	61
Marc et al. [21]	2021	DR Congo	89	89	64.4	26	63
Belayneh et al. [22]	2022	Ethiopia	131	40	N/A	24	16

Mean age was absent in one study [22]. The study with the highest sample size is 4,181 [17], while 31 is the smallest sample size in this study [10]. The study types included in this review are retrospective studies [9-18], cross-sectional studies [20,21], and case-control studies [22].

Identified risk factors

Table 2 below itemizes the risk factors identified by the included articles, including the number of patients with various risk factors. Deep vein thrombosis (DVT), heart disease, immobilization, obesity, smoking, recent surgery, and malignancy were the most commonly identified risk factors across the included articles. There was the presence of comorbidities such as diabetes, cardiac illness, HIV/AIDS, pulmonary tuberculosis (PTB), chronic obstructive pulmonary disease (COPD), and malignancy [13,16,17,20,22].

**Table 2:** Risk factors of PE. DVT: deep vein thrombosis; SCD: sickle cell disease; PTB: pulmonary tuberculosis; PE: pulmonary embolism

Risk factors	Number of patients
DVT	89
Immobilization	72
Malignancy	33
Cardiac diseases	15
Pregnancy/postpartum	24
Obesity	57
Recent hospitalization	29
Coagulation disorders	8
SCD	2
Trauma	27
Smoking	53
Contraceptive	11
Previous history of PE	21
PTB	43
Age (>65)	5
Postoperative/recent surgery	43
Long journey	18
Others	39

The most reported risk factors from the included study are as follows: immobilization, obesity, smoking, DVT, recent surgery, malignancy, and recent hospitalization.

Outcome of PE

Patients with PE diagnosed with the help of CTPA received some treatment during hospital admission which includes anticoagulants [10-13,17,19,21] which are the most common treatment received, thrombolytics [10,12,13,16,20,21], fibrinolytics [11], vena cava filter [10,16], elastic compression bandage [12], and mechanical ventilation [20]. The outcome includes recovery and then home discharge and mortality. The number of patients discharged home is more than the number of deceased as shown in Table 3 below. There was an absence of the type of treatment received in one of the articles [22], while three articles didn't mention the treatment and outcome of patients diagnosed with PE [14,15,18].

**Table 3:** Treatment received and outcome. CTPA: computed tomography pulmonary angiography

Author	Diagnostic criteria	Treatment received	Discharged home	Deceased
Ogunkoya et al. [10]	CTPA	Anticoagulant, thrombolytic, vena cava filter	28 (90%)	3 (10%)
Pessinaba et al. [11]	CTPA	Anticoagulant, fibrinolytic	44 (86%)	7 (14%)
Manuel et al. [12]	CTPA	Anticoagulant, thrombolytic, elastic compression bandage	22 (59%)	15 (41%)
Ogeng'o et al. [13]	CTPA	Anticoagulant, thrombolytic	92 (72%)	36 (28%)
Tambe et al. [14]	CTPA	N/A	N/A	N/A
Bulajic et al. [15]	CTPA	N/A	N/A	N/A
Meel et al. [16]	CTPA	Thrombolytic, inferior vena cava filter	119 (81%)	28 (19%)
Russell et al. [17]	CTPA	Anticoagulant	65 (82%)	14 (18%)
Hussein et al. [18]	CTPA	N/A	N/A	N/A
Bakebe et al. [19]	CTPA	Anticoagulant	54 (93%)	4 (7%)
Raghubeer et al. [20]	CTPA	Thrombolytic, embolectomy, mechanical ventilation	71 (88%)	10 (12%)
Marc et al. [21]	CTPA	Anticoagulant, thrombolytic	60 (67%)	29 (33%)
Belayneh et al. [22]	CTPA	N/A	39 (98%)	1 (2%)

Discussion

The results of this review align with global literature on PE but also highlight unique aspects within the African context. Demographic findings revealed a higher prevalence of PE among older adults, with a mean age range of 40.8-64.4 years, and a predominance among females. Similar trends have been observed internationally, where advanced age is consistently linked to increased PE risk due to diminished mobility, comorbidities, and weakened vascular function [2]. Additionally, the elevated incidence among women aligns with studies showing that hormonal influences, such as pregnancy and contraceptive use, increase thrombosis risk [23].

The review identifies DVT, immobilization, heart disease, obesity, smoking, recent surgery, and malignancy as common PE risk factors, consistent with findings in Europe and North America. A similar study in the United States reported that DVT and prolonged immobilization are primary PE risk factors, particularly following surgery or trauma [3,24]. In multivariate analysis, Goldhaber et al. identified obesity, cigarette smoking, and hypertension as independent predictors of PE especially among obese women and heavy cigarette smokers [25]. Morris et al. identified cesarean section as the major risk factor among postpartum women, while Knight identified multiparty, obesity, and a previous history of PE [26,27]. A Framingham study by Goldhaberet al. also identifies age (>43 years), smoking, obesity, and high blood pressure [28]. However, this review underscores unique regional factors, such as a heightened prevalence of PE in postpartum women and contraceptive users, likely due to healthcare access limitations and demographic differences in contraceptive use. Studies from other low- and middle-income regions have also reported increased PE risk in postpartum women, suggesting that limited access to antenatal care and thrombotic risk assessments could be contributing factors [1]. In contrast to other developed countries, Lee et al. identified recent surgery of less than three months, previous VTE, immobilization, malignancy, indwelling venous catheter, coagulopathy, and presence of infection as the most common risk factors [29,30].

The review's reliance on CTPA as the primary diagnostic tool for PE aligns with international guidelines. CTPA is regarded as the gold standard for PE diagnosis, but its limited availability in African settings creates challenges for timely and accurate diagnosis [17]. Studies from resource-constrained settings have shown that the absence of advanced diagnostic tools can lead to the underdiagnosis or misdiagnosis of PE, potentially increasing morbidity and mortality [20]. This diagnostic gap suggests an urgent need for affordable and portable diagnostic alternatives in low-resource environments.

Treatment practices in the review primarily involved anticoagulation therapy, with thrombolytics and vena cava filters used in selected cases, echoing global standards. However, inconsistent reporting of treatment specifics across the studies indicates disparities in healthcare resources and practices. This variability in treatment availability is similarly noted in studies from other resource-limited regions, which often rely on anticoagulation as the primary therapy due to cost and resource constraints [19]. Kovacs et al. treated inpatients and outpatients with anticoagulant therapy, and there was significant improvement in patient well-being [31]. In developed settings, however, thrombolytic and invasive treatments like vena cava filters are more widely available, improving patient outcomes [16]. Planer et al. compared the use of catheter-directed thrombolysis (CDT) with systemic thrombolysis and anticoagulation and highlighted that CDT was associated with a decreased risk of death, intracerebral hemorrhage, and any major bleeding [32-34].

Finally, patient outcomes in the review demonstrated a generally high rate of recovery and discharge, though mortality rates were significant in some cases. The unavailability of interventional treatment modality may cause increased mortality in some studies as studies have shown a significant reduction in mortality with the use of interventional treatment methods [32-34]. The presence of comorbidity, i.e., diabetes, cardiac illness, HIV/AIDS, PTB, COPD, and malignancy, was reported in our study and increased the likelihood of mortality in some patients [13,16,17,20,22], and a study by Eckelt et al. also reported this observation [35]. A case series by Goncalves et al. [36] and another study by Ambrosetti et al. [37] reported the association of PTB with the development of PE. According to the WHO, in 2022, 2.5 million people fell ill with TB in the African region, accounting for a quarter of new TB cases worldwide. An estimated 424,000 people died from the disease in the African region (1.267 million globally) in 2022 and over 33% of TB deaths occur in the African region [38]; 25.6 million people are living with HIV in the African region and about 380,000 people died from AIDS-related illness in 2022 [39].

Studies have also shown the association of HIV/AIDS with PE [40-42]. The lack of standardized outcome reporting limits comparability across studies and impedes the comprehensive assessment of treatment effectiveness and long-term outcomes. Similar limitations in outcome tracking have been documented in low-resource healthcare settings, where limited follow-up capacity and record-keeping practices often prevent long-term outcome analysis [18]. Additionally, the lack of longitudinal data in the studies limits the understanding of recurrence rates and chronic impacts, a gap noted in broader literature that advocates for more comprehensive follow-up of PE patients to guide post-discharge care [2].

Addressing inconsistencies and gaps

The review revealed inconsistencies in treatment and outcome reporting across studies, with some studies not specifying the use of anticoagulants or thrombolytics, likely reflecting resource constraints. Standardizing outcome measures and encouraging comprehensive reporting could enhance the understanding of treatment effectiveness and resource limitations in African healthcare settings [11,18,19]​.

Recommendations/future research directions

Genetic and Environmental Factors

Exploring genetic predispositions specific to African populations might reveal inherited thrombophilia or other conditions linked to PE. Genetic and environmental factor exploration could shed light on abnormalities unique to the African region, aiding in the development of targeted screening protocols and preventive care strategies [13]. Partnerships with NGOs in African countries could also play a vital role by promoting public awareness campaigns and investigating research to better understand how contraceptive use, postpartum factors, and genetic predispositions contribute to the risk of PE.

Infectious Disease Impact

Considering the high prevalence of HIV and TB, which influence coagulation, future research should investigate the interaction between these diseases and PE risk, potentially guiding specialized preventive measures [11].

Socioeconomic Barriers

Research into the impact of healthcare access on PE outcomes could inform policies that address inequities in resource distribution, a notable issue underscored by the inconsistency in diagnostic and treatment capabilities across African studies [18].

Long-Term Outcome/Longitudinal Studies

Most studies in this review were cross-sectional or retrospective, limiting insights into long-term outcomes for PE patients. Prospective cohort studies are needed to examine recurrence rates, mortality, and quality of life in African populations. Barriers to long-term studies could be resolved by the establishment of a regional and central health database through data collection through mobile devices and telemedicine for follow-up across the African continent. 

Prevention Protocols in Surgical and Postpartum Settings

With immobilization and postpartum status emerging as significant risk factors, research evaluating the efficacy of DVT and PE prevention protocols in African hospitals could provide data-driven improvements to clinical care [11,13,19].

International organizations support African regions by building standardized facilities, providing subsidies for laboratory equipment, and training medical doctors on the latest diagnostic and treatment guidelines and laboratory staff and engineers on the use of the equipment and facilitating its repair. The government of each country works to establish an effective healthcare system by employing qualified medical professionals, acquiring or subsidizing medical equipment for the diagnosis of PE in all government-owned hospitals, and improving access to federal or state health insurance schemes to cover the cost of investigation and treatments.

Study strengths and limitations

This study presents significant strengths and some notable limitations in its examination of PE risk factors and outcomes among African patients. One of its key strengths is the comprehensive regional focus, which provides much-needed insights into PE within African populations. Unlike many studies that offer general or Western-focused perspectives, this review highlights region-specific risk factors, such as the heightened prevalence of PE among postpartum women and contraceptive users. This localized approach addresses the gap in context-specific data for Africa and enables more targeted recommendations for clinical practices and healthcare policies tailored to regional needs.

The extensive data collection across diverse African countries adds to the robustness of the study’s findings. By reviewing data from 13 studies and covering over 7,600 patients, the study achieves a degree of generalizability across various African contexts. This breadth allows for the identification of both widely acknowledged risk factors, such as DVT and immobilization, and emerging concerns specific to the African population. Including rigorous inclusion and exclusion criteria further enhances the study's reliability by ensuring that only studies meeting defined quality standards, such as CTPA-confirmed PE diagnoses, were selected for analysis.

However, the study is also limited by several factors. Despite an initial pool of over 700 articles, only 13 studies met the stringent inclusion criteria, limiting the scope of analysis. This narrow selection means that some African countries, especially those with fewer research resources or lower PE documentation rates, are not represented. This gap might skew the data toward settings with stronger research outputs and potentially mask variations in PE prevalence or risk factors in less-represented regions.

Additionally, the study encompasses a mix of retrospective, cross-sectional, and case-control studies, which introduces methodological heterogeneity. Such variability complicates the synthesis of findings and may limit the ability to make definitive conclusions about causation. The reliance on retrospective data is another constraint, as retrospective studies are often hampered by issues like incomplete patient records and inherent biases. This reliance on past data may impact the accuracy of recorded outcomes and hinder a real-time understanding of PE risks.

The lack of standardized outcome reporting across the included studies also presents challenges. While the review successfully identifies common treatments, such as anticoagulants and thrombolytics, there is inconsistency in reporting treatment specifics. Some studies do not detail the types of therapies used, which makes it difficult to evaluate the effectiveness of different treatments across African healthcare settings. Standardized reporting would allow for a more accurate assessment of clinical outcomes and treatment efficacy.

Another limitation lies in the absence of longitudinal data, as most included studies do not provide follow-up on long-term patient outcomes, such as recurrence rates or post-PE quality of life. This lack of longitudinal analysis restricts insights into the chronic effects of PE and the benefits of long-term management strategies, which are critical for formulating comprehensive treatment protocols.

Finally, the study indirectly highlights the resource limitations faced by many African healthcare systems, particularly regarding access to advanced diagnostics like CTPA. Given the importance of accurate PE diagnosis, limited diagnostic resources may lead to underdiagnosis or misdiagnosis, thereby underestimating the true incidence of PE in the region. This diagnostic gap points to the need for affordable and accessible diagnostic alternatives to improve PE detection and management in low-resource settings.

## Conclusions

This systematic review provides essential insights into PE among African populations, identifying key risk factors such as DVT, immobilization, heart disease, obesity, smoking, recent surgery, and malignancy. The findings reveal unique demographic trends, such as heightened PE risk in postpartum women and contraceptive users, underscoring the need for tailored healthcare strategies. Limited access to advanced diagnostics like CTPA poses a significant challenge, contributing to underdiagnosis and treatment delays. Additionally, variability in treatment protocols, particularly in the use of anticoagulation and thrombolytics, highlights disparities in healthcare resources across the continent and calls for standardized, resource-sensitive clinical guidelines.

The review also identifies research gaps, notably the absence of prospective, longitudinal studies on long-term PE outcomes, such as recurrence and mortality. Future studies should explore the intersection of PE with prevalent conditions like HIV and TB, as well as genetic and environmental factors unique to Africa. Addressing these gaps through improved diagnostics, consistent treatment practices, and expanded research could enhance PE care, reduce morbidity, and improve patient outcomes across African healthcare settings.

## Appendices

Appendix A

**Table 4:** Search strategy. AJOL: African Journals Online

Search strategy	Database	Outcome
(pulmonary embolism) AND (Africa)	PubMed	301
"pulmonary embolism" "Africa"	Scopus	118
"pulmonary embolism" "Africa"	AJOL	300

Appendix B

**Table 5:** JBI critical appraisal tool for retrospective cohort studies. JBI: Joanna Briggs Institute; Y: yes, N: no

Checklist question	Ogunkoya et al. [10]	Pessinaba et al. [11]	Manuel et al. [12]	Ogeng'o et al. [13]	Tambe et al. [14]	Bulajic et al. [15]	Meel et al. [16]	Russell et al. [17]	Hussein et al. [18]	Bakebe et al. [19]
Were the two groups similar and recruited from the same population?	Y	Y	Y	Y	Y	Y	Y	Y	Y	Y
Were the exposures measured similarly to assign people to both exposed and unexposed groups?	Y	Y	Y	Y	Y	Y	Y	Y	Y	Y
Was the exposure measured in a valid and reliable way?	Y	Y	Y	Y	Y	Y	Y	Y	Y	Y
Were confounding factors identified?	Y	Y	Y	Y	Y	Y	Y	Y	Y	Y
Were strategies to deal with confounding factors stated?	Y	Y	Y	Y	Y	Y	Y	Y	Y	Y
Were the groups/participants free of the outcome at the start of the study (or at the moment of exposure)?	Y	Y	Y	Y	Y	Y	Y	Y	Y	Y
Were the outcomes measured in a valid and reliable way?	Y	Y	Y	Y	Y	Y	Y	Y	Y	Y
Was the follow-up time reported and sufficient to be long enough for outcomes to occur?	N	N	N	N	N	N	N	N	N	N
Was the follow-up complete, and if not, were the reasons for loss to follow-up described and explored?	N	N	N	N	N	N	N	N	N	N
Were strategies to address incomplete follow-up utilized?	N	N	N	N	N	N	N	N	N	N
Was appropriate statistical analysis used?	Y	Y	Y	Y	Y	Y	Y	Y	Y	Y

Appendix C

**Table 6:** JBI critical appraisal tool for cross-sectional studies. JBI: Joanna Briggs Institute; Y: yes

Checklist question	Raghubeer et al. [20]	Marc et al. [21]
Were the criteria for inclusion in the sample clearly defined?	Y	Y
Were the study subjects and the setting described in detail?	Y	Y
Was the exposure measured in a valid and reliable way?	Y	Y
Were objective, standard criteria used for the measurement of the condition?	Y	Y
Were confounding factors identified?	Y	Y
Were strategies to deal with confounding factors stated?	Y	Y
Were the outcomes measured in a valid and reliable way?	Y	Y
Was appropriate statistical analysis used?	Y	Y

Appendix D

**Table 7:** JBI critical appraisal tool for case-control studies. JBI: Joanna Briggs Institute; Y: yes

Checklist question	Belayneh et al. [22]
Were the groups comparable other than the presence of disease in cases or the absence of disease in controls?	Y
Were cases and controls matched appropriately?	Y
Were the same criteria used for the identification of cases and controls?	Y
Was exposure measured in a standard, valid, and reliable way?	Y
Was exposure measured in the same way for cases and controls?	Y
Were confounding factors identified?	Y
Were strategies to deal with confounding factors stated?	Y
Were outcomes assessed in a standard, valid, and reliable way for cases and controls?	Y
Was the exposure period of interest long enough to be meaningful?	Y
Was appropriate statistical analysis used?	Y
